# Analysis of Risk Factors for Patients with Liver Cirrhosis Complicated with Spontaneous Bacterial Peritonitis

**Published:** 2018-12

**Authors:** Yuan WANG, Qingyu ZHANG

**Affiliations:** Tianjin Medical University General Hospital, Tianjin 300052, China

**Keywords:** Liver cirrhosis, Spontaneous bacterial peritonitis, Risk factor, Bacterial culture

## Abstract

**Background:**

We aimed to explore risk factors for patients with liver cirrhosis complicated with spontaneous bacterial peritonitis (SBP).

**Methods:**

The clinical data of 195 patients with liver cirrhosis complicated with SBP (study group), admitted from March 2014 to March 2018 in Tianjin Medical University General Hospital, Tianjin, China and 195 patients without liver cirrhosis complicated with SBP (control group) were retrospectively analyzed. Clinical manifestations of patients and laboratory findings were analyzed. Univariate and multivariate Logistic regression analysis were used for independent risk factors for the occurrence of SBP.

**Results:**

There were significant differences in patients between study group and control group in Child-Pugh classification, peripheral blood white blood cell (WBC), serum C-reactive protein (CRP), serum total bilirubin (TBil), ascites WBC, ascites albumin (ALB), and the ratio of complicated with upper gastrointestinal hemorrhage, hepatorenal syndrome, hepatic encephalopathy and hyponatremia (*P*<0.01); Logistic regression analysis found that Child-Pugh classification, serum CRP, ascites WBC, ascites ALB, upper gastrointestinal hemorrhage, hepatorenal syndrome, hepatic encephalopathy and hyponatremia were related to the occurrence of SBP; and Child-Pugh classification, ascites ALB, upper gastrointestinal hemorrhage, hepatorenal syndrome and hyponatremia were its independent risk factors.

**Conclusion:**

Child-Pugh classification, ascites ALB, upper gastrointestinal hemorrhage, hepatorenal syndrome and hyponatremia are independent risk factors for the occurrence of liver cirrhosis complicated with SBP. Cautions should be raised for patients with liver cirrhosis for this. In the early stage, we should make effective antibiotic anti-bacterial infection program, actively prevent and control the occurrence of SBP, improving the survival rate of patients.

## Introduction

Liver cirrhosis is one of the common diseases in human beings, which is a diffuse liver damage caused by many factors for a long time (1). Liver cirrhosis is often accompanied by severe complications, and spontaneous bacterial peritonitis (SBP) is a common severe complication in patients with liver cirrhosis, which refers to a series of pathological symptoms, such as intestinal abscess and intestinal perforation induced by the infection of organs in the abdominal cavity (2). The incidence of SBP in patients with end-stage liver cirrhosis is about 10%∼30%. When liver cirrhosis occurs, the immunity of human body severely decreases, especially the phagocytosis of white blood cells (WBC) and macrophages in the body, thus destroying the stability of the hepatic endoplasmic reticulum endothelial system. With the prolongation of time, complications such as upper gastrointestinal hemorrhage, hepatic encephalopathy and hepatorenal syndrome can be induced. SBP is one of the leading causes of death in patients with liver cirrhosis (3–5).

The early clinical symptoms of most patients with SBP are not typical, the positive rate of ascites bacterial culture is low, and bacteria culture needs a certain period of time, so it is easy to cause missed diagnosis and misdiagnosis in clinic, which has an effect on the early treatment of patients (6). Nearly, 8%∼28% of patients with decompensated liver cirrhosis and 10%∼20% of patients with severe viral hepatitis are accompanied by SBP, the recurrence rate of which can reach 40% in 6 months, and it can reach 70% in one year. The mortality rate of patients with SBP for the first time can reach 20% and the mortality rate after one year is as high as 70%. The disease has high incidence, recurrence rate and mortality rate (7, 8).

At present, there are few studies on the risk factors for the occurrence and etiology of SBP, and the early effective diagnosis and treatment of SBP has not been clarified. Therefore, finding the etiology and risk factors closely related to the disease can identify the potential threat factors for the patients early, which is of great significance for the prevention, treatment and prognosis of the disease.

In this study, the clinical manifestations and laboratory findings of 195 patients with liver cirrhosis complicated with SBP and 195 patients without liver cirrhosis complicated with SBP were observed and analyzed, in order to provide clinical reference for the prevention of the occurrence of complications of SBP in patients with liver cirrhosis.

## Materials and Methods

### General Data

The clinical data of 195 patients with liver cirrhosis complicated with SBP (study group), admitted from March 2014 to March 2018 in Tianjin Medical University General Hospital, Tianjin, China and the clinical data of 195 patients without liver cirrhosis complicated with SBP (control group) were retrospectively analyzed.

This study was approved by the Ethics Committee of Tianjin Medical University General Hospital. Patients who participated in this research, signed the informed consent and had complete clinical data.

There were 149 males and 46 females in study group, and the age range was 21∼85 yr old, with an average age of 50.2±11.8 yr old. There were 132 males and 63 females in control group, and the age range was 23∼84 yr old, with an average age of 51.7±10.6 years old.

Inclusion criteria: With a history of chronic liver disease, the clinical symptoms and imaging tests were in accordance with the diagnostic criteria for liver cirrhosis (9); the ascites polymorphic nucleus WBC count in study group was higher than 0.25×109/L. Study group had typical clinical manifestations of peritonitis with fever, tenderness and abdominal pain in varying degrees.

Exclusion criteria: Patients with blood pressure of less than 90/60 mm Hg; patients who had been treated with antibiotics, microecological preparation therapy or abdominal operation four weeks before admission; patients complicated with severe cardiac dysfunction; patients complicated with primary liver cancer, tuberculous or secondary peritonitis; patients complicated with respiratory tract infection and urinary tract infection; patients with a previous history of mental illness and a family history of mental illness.

### Methods

The sex, age, cause of onset of the disease, Child-Pugh classification, peripheral blood red blood cell (RBC), peripheral blood WBC, peripheral blood platelet (PLT), peripheral blood hemoglobin (Hb), serum C-reactive protein (CRP), serum alanine aminotransferase (ALT), serum albumin (ALB), serum total bilirubin (TBil), ascites WBC, ascites ALB, upper gastrointestinal hemorrhage or not, hepatorenal syndrome or not, diarrhea or not, hepatic encephalopathy or not, hyponatremia or not and diabetes mellitus or not in patients in study group and control group were recorded. The data was counted and the risk factors for the occurrence of SBP were analyzed. The ascites of the patient in study group were etiologically identified. Bacterial culture strictly obeyed the principle of sterile operation, and 10 mL of ascites from the patient was taken to be etiologically identified by BD Phoenix Automatic Microbiological Analysis System (Guangzhou Ximark Biotechnology Co., Ltd), and corresponding anti-bacterial treatment was given.

### Statistical Methods

PSS19.0 (Analysis Software (Shanghai) Co., Ltd.) was used for statistical analysis. The measurement data was expressed by mean ± standard deviation (x̄±s). The counting data between groups were tested by chi-square, and the measurement data between groups were tested by *t* test. The risk factors for the occurrence of SBP were analyzed by Logistic single factor and multivariate regression. When *P*<0.05, the difference was statistically significant.

## Results

### Relationships between Clinical Data and SBP in Patients in Study Group and Control Group

There was no difference in sex, age, cause of onset of the disease, peripheral blood RBC, peripheral blood PLT, peripheral blood Hb, serum ALT and serum ALB in patients between study group and control group, which was not statistically significant. There was a significant difference in Child-Pugh classification, peripheral blood WBC, serum CPR, serum TBil, ascites WBC and ascites ALB in patients between study group and control group (*P*<0.01) (Table 1).

**Table 1:** Relationships between Clinical Data and SBP in Patients in Study Group and Control Group [n (%)] (x±s)

***Category***	***Study Group (n=195)***	***Control Group (n=195)***	***t/χ^2^***	***P***
Sex			3.680	0.071
Male	149 (76.41)	132 (67.69)		
Female	46 (23.59)	63 (32.31)		
Age (yr)	50.2±11.8	51.7±10.6	1.321	0.187
Cause of Onset of the Disease			0.420	0.810
Viral Liver Cirrhosis	143 (73.33)	139 (71.28)		
Alcoholic Liver cirrhosis	35 (17.95)	40 (20.51)		
Primary Biliary Liver Cirrhosis	17 (8.72)	16 (8.21)		
Child-Pugh Classification			15.386	0.001
Grade A	12 (6.15)	26 (13.33)		
Grade B	55 (28.21)	78 (40.00)		
Grade C	128 (65.64)	91 (46.67)		
Peripheral Blood RBC (×1012/L)	4.5±0.6	4.4±0.5	1.788	0.074
Peripheral Blood WBC (×109/L)	3.9±0.8	5.3±1.3	12.810	0.001
Peripheral Blood PLT (×109/L)	143.7±29.4	148.7±32.8	1.585	0.113
Peripheral Blood Hb (g/L)	131.8±4.9	132.7±4.5	1.889	0.059
Serum CRP (μg/ml)	73.8±19.6	24.7±8.4	32.150	0.001
Serum ALT (U/L)			1.468	0.289
≥Twice the Normal Value	21 (10.77)	29 (14.87)		
< Twice the Normal Value	174 (89.23)	166 (85.13)		
Serum ALB (g/L)			2.532	0.137
≥25	134 (68.72)	119 (61.03)		
<25	61 (31.28)	76 (38.97)		
Serum TBil (μmol/L1)			10.232	0.002
≤51.8	143 (73.33)	113 (57.95)		
>51.8	52 (26.67)	82 (42.05)		
Ascites WBC (/mm^3^)			16.894	0.001
≥500	134 (68.72)	94 (48.21)		
<500	61 (31.28)	101 (51.79)		
Ascites ALB (g/L)			8.135	0.006
≥10	73 (37.44)	101 (51.79)		
<10	122 (62.56)	94 (48.21)		

### Relationships between Complications and SBP in Patients in Study Group and Control Group

There was no difference in the complications of diarrhea and diabetes mellitus in patients between study group and control group, which was not statistically significant. There was a significant difference in the complications of upper gastrointestinal hemorrhage, hepatorenal syndrome, hepatic encephalopathy and hyponatremia in patients between study group and control group (*P*<0.01) (Table 2).

**Table 2:** Relationships between Complications and SBP in Patients in Study Group and Control Group [n (%)]

***Complications***	***Study Group (n=195)***	***Control Group (n=195)***	***Χ^2^***	***P***
Upper Gastrointestinal Hemorrhage			14.099	0.001
Yes	38 (19.49)	13 (6.67)		
No	157 (80.51)	182 (93.33)		
Hepatorenal Syndrome			27.841	0.001
Yes	31 (15.90)	2 (1.03)		
No	164 (84.10)	193 (98.97)		
Diarrhea			0.510	0.504
Yes	5 (2.56)	3 (1.54)		
No	190 (97.44)	192 (98.46)		
Hepatic Encephalopathy			21.902	0.001
Yes	28 (14.36)	3 (1.54)		
No	167 (85.64)	192 (98.46)		
Hyponatremia			13.595	0.001
Yes	67 (34.36)	35 (17.95)		
No	128 (65.64)	160 (82.05)		
Diabetes Mellitus			2.353	0.159
Yes	32 (16.41)	44 (22.56)		
No	163 (83.59)	151 (77.44)		

### Analysis of Risk Factors for Liver Cirrhosis Complicated with SBP

Through Logistic single factor analysis of risk factors associated with liver cirrhosis complicated with SBP, it was found that Child-Pugh classification (*P*=0.001), serum CRP (*P*=0.005), ascites WBC (*P*=0.001), ascites ALB (*P*=0.001), upper gastrointestinal hemorrhage (*P*=0.001), hepatorenal syndrome (*P*=0.002), hepatic encephalopathy (*P*=0.001) and hyponatremia (*P*=0.001) in patients were related to the occurrence of SBP (Table 3).

**Table 3:** Logistic Single Factor Analysis of Risk Factors for Liver Cirrhosis Complicated with SBP

***Clinical Parameters***	***Coefficients***	***Standard Errors***	***Wald Value***	***P Value***	***OR Value***	***95% CI***
Child-Pugh Classification	0.342	0.107	11.117	0.001	4.537	1.568–8.667
Peripheral Blood WBC (×109/L)	1.140	0.600	3.627	0.057	3.128	0.963–10.127
Serum CRP (μg/ml)	1.511	0.535	7.991	0.005	4.535	1.591–12.936
Serum TBil (μmol·L-1)	1.121	0.610	3.372	0.068	3.067	0.928–10.142
Ascites WBC (/mm3)	2.354	0.422	31.309	0.001	10.547	4.621–24.073
Ascites ALB (g/L)	2.159	0.448	19.558	0.001	8.664	3.328–22.563
Upper Gastrointestinal Hemorrhage	0.624	0.192	10.41	0.001	1.867	1.278–2.729
Hepatorenal Syndrome	1.327	0.423	9.943	0.002	3.770	1.632–8.537
Hepatic Encephalopathy	1.933	0.357	30.298	0.001	7.215	3.573–14.557
Hyponatremia	0.457	0.146	11.134	0.001	3.542	1.257–5.821

In order to control the influences of confounding factors on the results, risk factors related to the occurrence of SBP were introduced into Logistic multivariate regression analysis.

Child-Pugh classification, ascites ALB, upper gastrointestinal hemorrhage, hepatorenal syndrome and hyponatremia were independent risk factors for the occurrence of SBP (Table 4).

**Table 4:** Logistic Multivariate Analysis of Risk Factors for Liver Cirrhosis Complicated with SBP

***Clinical Parameters***	***Coefficients***	***Standard Errors***	***Wald Value***	***P Value***	***OR Value***	***95% CI***
Child-Pugh Classification	0.447	0.497	9.453	0.001	1.563	0.624–2.043
Serum CRP (μg/ml)	1.083	0.643	2.765	0.098	2.924	0.824–10.367
Ascites WBC (/mm3)	0.583	0.645	4.079	0.071	1.834	1.576–3.473
Ascites ALB (g/L)	2.347	0.401	13.537	0.001	10.455	4.765–21.943
Upper Gastrointestinal Hemorrhage	0.881	0.229	15.661	0.001	2.437	1.567–3.728
Hepatorenal Syndrome	0.741	0.418	21.336	0.047	2.096	1.923–4.757
Hepatic Encephalopathy	0.524	0.457	4.425	0.067	1.563	0.624–2.109
Hyponatremia	3.354	0.836	15.842	0.001	21.863	6.793–35.613

### Distribution and Treatment of Pathogenic Bacteria in Patients with Liver Cirrhosis Complicated with SBP

The results of etiological examination showed that of 195 patients with liver cirrhosis complicated with SBP, the results of bacterial culture were positive in 26 cases, and the positive rate was 13.33%. Overall, 32 strains of pathogenic bacteria were detected, including 18 (56.25%) Gram-negative bacteria strains, 12 (37.50%) Gram-positive bacteria strains, and 2 (6.25%) fungi strains (Table 5). Based on the treatment of primary diseases, corresponding anti-bacterial treatment was given to patients with liver cirrhosis complicated with SBP about the result of bacterial culture. The main drugs were cephalosporins and quinolones. After the treatment of 195 patients, 76 cases (38.97%) were improved or cured, 96 cases (49.23%) were ineffective or deteriorated, and 23 cases (11.79%) died.

**Table 5:** Distribution and Strain Composition Ratio of Pathogenic Bacteria in Patients with Liver Cirrhosis Complicated with SBP

***Pathogenic Bacteria***	***Strain number***	***Strain Composition Ratio (%)***
**Gram-negative Bacteria**	18	56.25
Escherichia Coli	8	25.00
Klebsiella Pneumoniae	4	12.50
Aeromonas	3	9.38
Pseudomonas Aeruginosa	1	3.13
Enterobacter Cloacae	2	6.25
**Gram-positive Bacteria**	12	37.50
Staphylococcus Epidermidis	8	25.00
Streptococcus	3	9.38
Enterococcus	1	3.13
**Fungus**	2	6.25
Candida Glabrata	1	3.13
Candida Albicans	1	3.13

## Discussion

SBP is a common severe complication in patients with liver cirrhosis. It is intra-abdominal infection mainly caused by pathogenic bacteria, blood or lymphatic system, mainly found in patients with severe liver disease or advanced liver cirrhosis, and it is characterized by rapid progress and high mortality (10,11). The patients with liver cirrhosis complicated with SBP have low immunity and long-term infection in the abdominal cavity, which weakens the functions of phagocytes in their own livers, resulting in the decrease of body resistance and the occurrence of a series of complications, which increases the mortality rate of patients (12). At present, the pathogenesis of SBP has not been clarified, and most scholars think that it is related to the imbalance of intestinal bacteria growth and distribution. The abnormal bile secretion of patients with liver cirrhosis causes the intestinal tract to be invaded by external bacteria, which breaks the balance of the original normal intestinal flora. In addition, the patient’s resistance is weakened, and the concentrations of phagocytes, ascites and serum ALB decrease, and the ability to remove external bacteria is inadequate. All of these lead to the translocation and proliferation of pathogenic bacteria, which ultimately results in the occurrence of SBP (13). Liver cirrhosis complicated with SBP has a rapid progression, with a mortality of 20% (14). Once a patient with liver cirrhosis has SBP, it indicates that the disease has entered the end stage. Therefore, the investigation of clinical risk factors associated with liver cirrhosis complicated with SBP has important clinical values for early detection and prevention of SBP, for the improvement of the therapeutic effects of SBP and for the decrease of mortality rate.

In this study, the clinical data of the two groups of patients were analyzed retrospectively. Accordingly, there were significant differences between the two groups in Child-Pugh classification, peripheral blood WBC, serum CRP, serum TBiL, ascites WBC, ascites ALB, upper gastrointestinal hemorrhage, hepatorenal syndrome, hepatic encephalopathy and hyponatremia. Logistic single factor analysis found that Child-Pugh classification, serum CRP, ascites WBC, ascites ALB, upper gastrointestinal hemorrhage, hepatorenal syndrome and hepatic encephalopathy of the patient were related to liver cirrhosis complicated with SBP.

The results of this study suggested that Child-Pugh classification was one of the independent risk factors for the occurrence of liver cirrhosis complicated with SBP. Liver function Child-Pugh classification was an important index of liver function damage, and the lower the grade was, the worse the liver reserve function of the patient was. The functions of reticular cells, endothelial cells and macrophages decreased, and bacterial migration and bacteremia were prone to occur in patients, which indirectly increased the possibility of the occurrence of SBP (15). The lower the Child-Pugh classification was, the worse the immunity of the patient was (16). The level of body conditioning was low, and the conditions of intestinal congestion and portosystemic shunt were serious, which made translocation bacteria easier to invade into the abdominal cavity and blood, eventually leading to infection. Therefore, the Child-Pugh classification for liver cirrhosis could predict the occurrence of SBP, and more attentions should be paid to Child Grade C patients. The occurrence of SBP was also closely related to the ability of ascites in the abdominal cavity to clear pathogenic bacteria, and the function and activity of ascites depended on the content and the level of complement of ascites protein. The decrease of ascites protein would not only decrease the ability of ascites to clear pathogenic bacteria, but also led to the occurrence of SBP (17). The results of this study suggested that ascites ALB was another independent risk factor for liver cirrhosis complicated with SBP, consistent with another study (18). The probability of ascites ALB concentration being lower than that of 10 g/L patients with liver cirrhosis complicated with SBP was 9 times higher than that of ascites ALB concentration being higher than that of 10 g/L, suggesting that the decrease of ascites protein concentration was a risk factor for SBP.

We found that upper gastrointestinal hemorrhage was another independent risk factor for liver cirrhosis complicated with SBP. We found that liver cirrhosis complicated with upper gastrointestinal hemorrhage was caused by portal hypertension. When the portal vein pressure increased, bleeding, ischemia, hypoxia and congestion were prone to occur in intestinal tract, resulting in the increase of intestinal mucosal permeability. The intestinal environment was changed caused by damaged intestinal barrier, and the intestinal flora was disordered and shifted, which aggravated the severity of ascites. The inflammatory factors in ascites were further diluted, the concentration and complement level of protein decreased, and the immune function of the body decreased. As a result, abdominal infection was difficult to control, and repeated infection led to further damage of intestinal mucosa in patients with upper gastrointestinal hemorrhage, thus inducing the occurrence of SBP (19). Hyponatremia was more likely to occur in patients with decompensated liver cirrhosis. Hyponatremia often reflected the state of liver function in patients, and the lower the level of serum sodium was, the more severe the damage of liver function was. Hyponatremia could cause cell edema, which in turn led to a decrease in extracellular fluid levels, resulting in a decrease in blood volume and even brain edema, which was also a precursor of hepatorenal syndrome. With pathological ischemia of the intestine and liver, the intestinal mucosal permeability increased, causing abdominal infection and the occurrence of SBP (20).

In this study, it was found that hyponatremia and hepatorenal syndrome were also independent risk factors for the occurrence of liver cirrhosis complicated with SBP. Another study (21) also found that hyponatremia was an independent risk factor for SBP. Patients with decompensated liver cirrhosis had the highest risk of developing SBP when serum sodium was higher than 125 mmol/L.

The results of this study suggested that of 195 patients with liver cirrhosis complicated with SBP, the results of bacterial culture were positive in 26 cases, and the positive rate was 13.33%. The positive rate of pathogenic bacteria culture in ascites was low. Among them, Gram-negative bacteria were mainly *Escherichia coli* and Gram-positive bacteria were mainly *Staphylococcus* epidermidis. Based on the treatment of primary diseases, corresponding treatment was given to the patient about the result of bacterial culture, and the main drugs were cephalosporins and quinolones. After the treatment of 195 patients, 76 cases were improved or cured, 96 cases were ineffective or deteriorated, and 23 cases died. Treatment of the disease should focus on the resistance to *E. coli* and *S. epidermidis*.

In this study, the subjects were screened strictly according to the inclusion and exclusion criteria, which ensured the reliability of the study. However, this study did not discuss the prognostic risk factors in patients with liver cirrhosis complicated with SBP, and failed to provide a more scientific and effective prognostic prediction method for clinical work, so it had some limitations. Therefore, in the next study, we should analyze the data in this aspect, and further verify the conclusions of this study.

## Conclusion

There are many risk factors for liver cirrhosis complicated with SBP, and Child-Pugh classification, ascites ALB, upper gastrointestinal hemorrhage, hepatorenal syndrome and hyponatremia are independent risk factors for the occurrence of liver cirrhosis complicated with SBP. Cautions should be raised for patients with liver cirrhosis for this. In the early stage, we should make effective antibiotic anti-bacterial infection program, actively prevent and control the occurrence of SBP, and improve the survival rate of patients.

## Ethical considerations

Ethical issues (Including plagiarism, informed consent, misconduct, data fabrication and/or falsification, double publication and/or submission, redundancy, etc.) have been completely observed by the authors.
